# Robust Cell-Level Classification for Liquid-Based Cervical Cytology Using Deep Transfer Learning: A Multi-Source Study Addressing Scanner-Induced Domain Shifts

**DOI:** 10.3390/bioengineering13030289

**Published:** 2026-02-28

**Authors:** Gulfize Coskun, Mustafa Caner Akuner, Erkan Kaplanoglu

**Affiliations:** 1 Department of Mechatronics Engineering, Marmara University, 34722 Istanbul, Türkiye; akuner@marmara.edu.tr; 2 Department of Engineering Management and Technology, The University of Tennessee at Chattanooga, Chattanooga, TN 37403, USA; erkan-kaplanoglu@utc.edu

**Keywords:** deep learning, transfer learning, cervical cancer, Pap smear, whole slide imaging, liquid-based cytology

## Abstract

Automated analysis of liquid-based cervical cytology is increasingly supported by digital microscopy and deep learning. However, model generalization remains challenging due to scanner- and laboratory-induced domain shifts affecting color, texture, and morphology. In this study, we present a robust cell-level classification framework for liquid-based Pap smear cytology based on deep transfer learning, designed to operate under heterogeneous acquisition conditions. We construct a multi-source dataset by integrating three widely used public reference repositories (SIPaKMeD, Herlev, CRIC Cervix) with a proprietary cohort comprising 416 Whole Slide Images (WSIs) collected from two medical centers and digitized using different scanning systems. All labels are harmonized into four Bethesda categories (NILM, ASC-US, LSIL, HSIL), and cell-centered 224 × 224 patches are used as standardized inputs for model development and benchmarking. We evaluate state-of-the-art CNN backbones (ResNet50, EfficientNetB0, VGG16) and perform systematic ablation across data-source combinations to quantify robustness under acquisition variability. Among the evaluated models, ResNet50 yields the best overall performance on the independent test set (accuracy = 0.91; macro-F1 = 0.91), consistently outperforming EfficientNetB0 and VGG16. Importantly, incorporating proprietary multi-center WSI-derived data improves robustness to scanner-induced variation compared to training on public data alone. These findings demonstrate that combining diverse data sources can mitigate domain shift in cell-level cervical cytology classification. While clinically actionable screening requires slide-level aggregation (e.g., MIL-based WSI inference), the proposed classifier provides a robust component that can be integrated into end-to-end WSI screening pipelines in future work.

## 1. Introduction

Cervical cancer remains a major global health burden despite being largely preventable through vaccination, screening, and timely treatment. Recent global estimates continue to report hundreds of thousands of new cases and deaths annually, with disproportionate impact in regions where organized screening and follow-up pathways are limited [1,2]. Cytology-based screening supported by liquid-based cytology (LBC) and standardized reporting via the Bethesda System has contributed substantially to reducing morbidity and mortality by enabling early identification of precursor lesions and risk stratification [3]. Nevertheless, manual cytology workflows are resource-intensive and subject to intra- and inter-observer variability, particularly for borderline and low-prevalence abnormalities, and the workload scales poorly in high-throughput screening environments [4,5]. These constraints have accelerated interest in digital cytopathology and computer-aided diagnosis systems as a means to improve efficiency, consistency, and overall screening performance. Over the past decade, deep learning has become the leading approach for medical image analysis, demonstrating strong performance in detection, segmentation, and classification tasks. In cervical cytology, many early deep learning studies were developed using single-cell or cropped cell-cluster images because such data are easier to annotate and computationally tractable. Public datasets such as Herlev and SIPaKMeD played a key role in enabling reproducible benchmarking and rapid algorithm development [6,7]. However, a recurring limitation identified across recent surveys and systematic reviews is that high performance on small, curated datasets often fails to translate to real-world clinical settings, where variability in preparation protocols, staining, and imaging devices is substantial [4,5,8]. This translation gap is especially pronounced when moving from cropped images to Whole Slide Imaging, where diagnostically relevant abnormalities are sparse and distributed across gigapixel-scale slides. WSI-based cervical cytology introduces distinct technical and clinical challenges. Unlike histopathology, where tissue architecture provides continuous regional context, cytology WSIs contain discrete cell objects, frequent overlap and clutter, heterogeneous backgrounds and scanning artifacts. Screening requires reliably identifying rare abnormal cells among tens of thousands of normal cells and artifacts, then aggregating evidence into a slide-level decision aligned with clinical categories [9]. Consequently, many modern WSI screening pipelines adopt a two-stage design, candidate localization of suspicious cells or regions and slide-level aggregation using learned or engineered statistics [9,10,11]. While these approaches have shown strong results in controlled settings, their robustness under routine multi-site variability remains a key barrier to clinical deployment.

A principal cause of limited deployability is domain shift systematic differences between training and deployment data distributions caused by site-specific staining protocols, slide preparation, scanner optics/sensors, compression, and color calibration. Domain shift can lead to substantial performance degradation when models encounter unseen scanners or laboratories, because models may learn spurious correlations that are stable within a single domain but not across domains [12]. In computational pathology, domain shift has been quantitatively characterized and shown to be a major contributor to generalization failure; stain normalization and strong augmentation can mitigate but not fully eliminate cross-domain performance drops [12,13]. In cervical cytology WSI screening, the impact can be even more pronounced because diagnostically relevant cues are subtle such as nuclear chromatin patterns, cytoplasmic staining characteristics, and the nucleus-to-cytoplasm ratio and may be confounded by differences in slide preparation, staining protocols, and scanner acquisition settings. Recent multi-center, large-scale studies have explicitly reframed robust generalization as the central requirement for AI-assisted cytology screening. STRIDE (Scalable Technology for Robust and Interpretable Diagnosis built on Extensive Data) proposes an end-to-end learning strategy that combines patient/slide-level supervision with a limited fraction of cell-level labels to overcome annotation bottlenecks, and introduces color-adversarial sample training to mimic staining and imaging variations for improved robustness to real-world domain shifts [14]. STRIDE is evaluated at unprecedented scale across 183 medical centers and hundreds of thousands of WSIs, emphasizing that robustness and interpretability are essential for trustworthy clinical integration [14]. Complementarily, Smart-CCS introduces a paradigm that couples large-scale self-supervised pretraining with test-time adaptation to improve generalizability in complex clinical scenarios. To support this, Smart-CCS is developed and validated on CCS-127K (127,471 cytology WSIs from 48 centers), demonstrating high performance across internal, external, and prospective cohorts and highlighting the role of pretraining and adaptation for stabilizing performance under unseen acquisition conditions [15]. Together, these works illustrate a field-wide shift: from small homogeneous benchmarks to multi-center WSI cohorts, and from purely supervised learning to hybrid strategies. Despite these advances, many institutions and applied research settings still face practical constraints: access to national-scale datasets is limited, annotation resources remain scarce, and scanner heterogeneity is the norm rather than the exception. Therefore, a pragmatic route toward clinical feasibility is to design datasets and evaluations that explicitly reflect real-world heterogeneity, particularly scanner-induced variability while employing robust training protocols. In this study, we address this need by constructing a multi-source dataset that combines standard public repositories (Herlev, SIPaKMeD, CRIC Cervix) with a proprietary multi-center cohort of 416 liquid-based cytology WSIs collected from two distinct medical centers (private and public) and digitized using different scanning systems. Using transfer learning with widely adopted CNN backbones (ResNet50, EfficientNetB0, VGG16), we quantify generalization under scanner-induced domain shifts and demonstrate the contribution of multi-center proprietary WSI data to robustness. By aligning our motivation with recent large-scale evidence on domain variability and generalizability our work aims to provide an actionable and reproducible pathway for developing cervical cytology AI systems that are closer to clinical deployment.

## 2. Materials and Methods

### 2.1. Dataset Composition and Acquisition

A retrospective study design was employed to develop a deep learning framework toward AI-assisted cervical cytology screening, focusing on the cell-centered classification component. To enhance generalization across heterogeneous data sources and mitigate scanner- and laboratory-induced domain shift, we combined established open-access benchmarks with a proprietary multi-center WSI-derived dataset.

#### 2.1.1. Public Reference Datasets

- To establish a reproducible baseline and to leverage well-characterized cytomorphological reference material, we incorporated open-access public datasets that provide expert-labeled cervical cytology images at the cell or cell-cluster level. These repositories are widely used for controlled benchmarking and feature learning. However, they do not capture the full heterogeneity of large-scale multi-center WSI screening cohorts. Recent state-of-the-art systems such as STRIDE [14] and Smart-CCS/CCS-127K [15] demonstrate WSI-scale screening under broad multi-center variability using large proprietary cohorts and robustness strategies. In this work, public reference datasets are therefore used to enrich morphological feature extraction and to support controlled comparisons, while scanner-induced variability is addressed through the inclusion of our proprietary multi-center WSI-derived cohort and corresponding ablation analyses. For this study, all class labels were remapped to the four standard Bethesda System categories (NILM, LSIL, HSIL, ASC-US) to create a unified training distribution:
- Herlev Dataset: Consists of 917 single-cell images acquired at high magnification (0.201 μm/pixel). The original labels follow a 7-class WHO system (Superficial, Intermediate, Columnar, Mild/Mod/Severe Dysplasia, Carcinoma in Situ). For this study, we remapped these into the Bethesda system. Although these are isolated cells with clean backgrounds, they serve as a gold standard for morphological feature extraction [8]. The Herlev Dataset was downloaded from http://mde-lab.aegean.gr/index.php/downloads (accessed on 4 March 2021).
- SIPaKMeD: Contains 4049 images of clustered cells manually cropped from CCD camera frames (966 × 966 pixels). Unlike Herlev, these images include background cytoplasm and overlapping nuclei, simulating a more realistic field of view [7]. SIPaKMeD Dataset was downloaded from https://www.cs.uoi.gr/~marina/sipakmed.html (accessed on 13 April 2021). The dataset comprising benign and dysplastic cell clusters was obtained from the SIPaKMeD Database available at the University of Ioannina repository.
- CRIC Cervix: is a searchable image database consisting of 400 images (1376 × 1020 pixels) from conventional Pap smears, featuring 11,534 manually classified cells. Each cell class is marked with a bounding box within these images. The class labels are stored in a separate classifications.csv file. A Python script was written to automate the cell extraction process: it reads the coordinate data, determines the cell’s class, and saves the cropped cell as a patch into its respective class folder [11]. CRIC Cervix Dataset was downloaded from https://www2.decom.ufop.br/cric/cric-database (accessed on 23 October 2024). Notably, our public reference component includes CRIC Cervix, ensuring that the baseline reference material is not limited to pre-2021 resources.

Since public datasets (Herlev) focus on centered single cells while WSI patches contain complex scenes, all inputs were spatially normalized. We treated the WSI patches as regions of interest (ROI) comparable to the clustered views in SIPaKMeD. All class labels were mapped to the four Bethesda categories [3] (NILM, LSIL, HSIL, ASC-US) to create a unified training distribution.

#### 2.1.2. Proprietary Multi-Center Dataset

To overcome the limitations of curated public data and bridge the gap between experimental benchmarking and clinical reality, a large-scale proprietary dataset comprising 416 Whole Slide Images (WSIs) was constructed. Crucially, each slide corresponds to a unique patient (*n* = 416) to strictly prevent patient-level data leakage during train/test splitting. Samples were retrospectively collected from two geographically and structurally distinct institutions in Türkiye to ensure broad demographic representation. Center A, a public university hospital, represents a patient population characterized by lower socio-economic status (SES), higher multiparity rates, and varying health literacy factors often correlating with advanced pathological stages at initial screening [16]. In contrast, Center B is a private clinic serving a cohort with higher SES and an urban lifestyle, presenting distinct lifestyle-associated risk factors. This dual-center approach captures not only technical variations but also biological variability stemming from diverse epidemiological backgrounds.

All slides underwent standard Papanicolaou staining to maintain diagnostic consistency. However, preparatory variability was introduced by including Liquid-Based Cytology (LBC) slides prepared using both ThinPrep (Hologic, Inc., Marlborough, MA, USA; *n* = 198) and BD SurePath (Becton, Dickinson and Company, Franklin Lakes, NJ, USA; *n* = 218) techniques [17,18]. To simulate realistic deployment scenarios, digitization was performed using three distinct high-throughput systems: the Leica Aperio GT 450 (Leica Biosystems Imaging, Inc., Vista, CA, USA), Hamamatsu NanoZoomer S60 (Hamamatsu Photonics K.K., Hamamatsu, Shizuoka, Japan), and 3DHistech Pannoramic 250 Flash (3DHISTECH Kft., Budapest, Hungary). While all scanners operated with a uniform Numerical Aperture (NA) of 0.95 to ensure consistent optical resolution, the multi-scanner approach introduced necessary variations in color temperature, sensor noise, and contrast.

To evaluate the model’s performance on unseen data, an independent test set comprising 120 WSIs was curated. This cohort was stratified to ensure class balance, consisting of 30 WSIs for each of the four diagnostic categories (NILM, LSIL, HSIL, ASC-US). From these slides, a total of 8763 single-cell patches were extracted and utilized for the final quantitative testing. This diversity forces the deep learning model to learn robust, scanner-agnostic features rather than overfitting to specific device characteristics [19].

**Figure 1:**
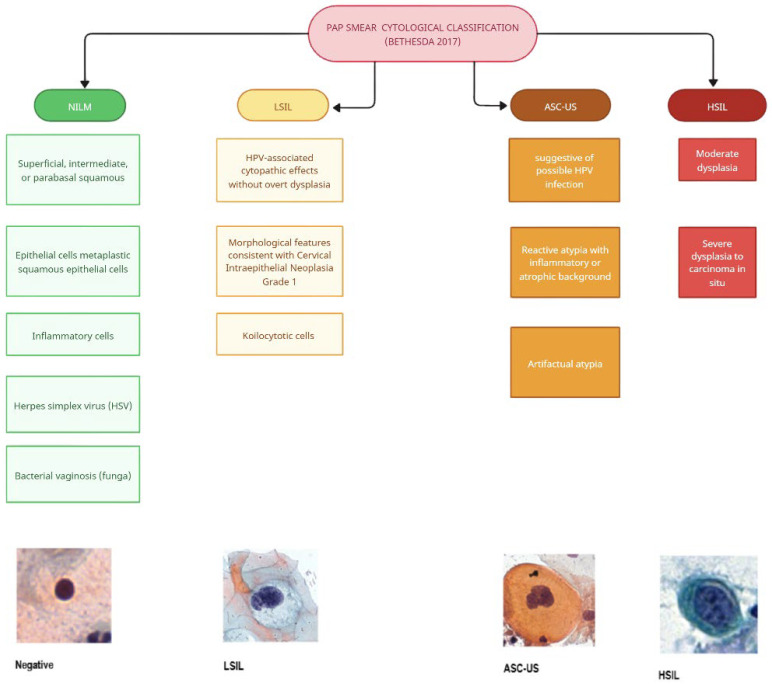
Cytological Classification of Pap Smear based on the Bethesda System: Hierarchical schematic of the Bethesda-based cytological classification applied to all Pap smear image patches. The four main diagnostic categories (green: NILM, yellow: LSIL, red: HSIL, orange: ASC-US) and their respective subclasses guided both manual expert labeling and supervised AI model training.

Overall, for the quantitative analysis in this study, a total of 13,090 single-cell image samples were used. The training set comprised 10,026 cells (Negative/NILM: 6512; HSIL: 2036; LSIL: 844; ASC-US: 634). The validation and test sets were initially identical in composition and contained 1532 cells each (Negative/NILM: 1793; ASC-US: 260; HSIL: 662; LSIL: 349), corresponding to 3064 samples in total across both splits. All Pap smear image patches were categorized according to the Bethesda 2017 cytological framework (Figure 1).

### 2.2. Data Preprocessing

Efficient data preprocessing is a critical prerequisite for handling gigapixel-scale Whole Slide Images (WSIs) within a Deep Learning framework [20]. To transform the raw digital scans into model-compatible inputs and ensure high data quality, a systematic processing pipeline was implemented. Specifically, the proprietary dataset was digitized at 40× magnification to capture fine-grained cellular morphology essential for cytopathological diagnosis. The subsequent workflow is structured into three distinct phases: (1) Annotation of Regions of Interest (ROIs) to define ground truth, (2) a strategic Patching process to tile WSIs into fixed-size tensors, and (3) Stain Normalization to mitigate scanner-induced color variations and harmonize the domain distribution [21] across different centers.

**Figure 2:**
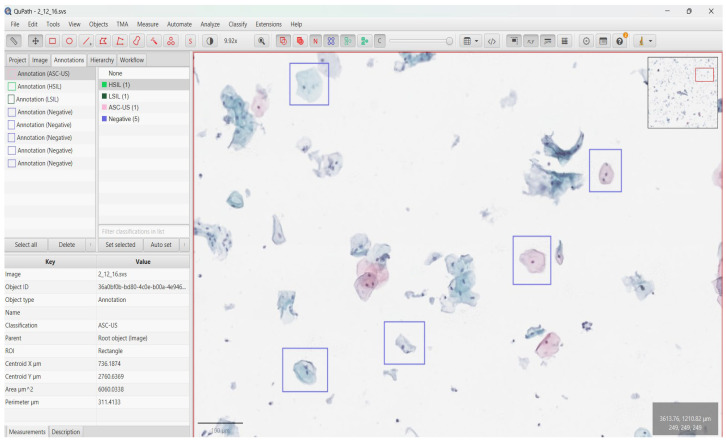
Expert-driven annotation and data extraction workflow using QuPath. Experienced cytopathologists manually reviewed WSIs and annotated representative single-cell regions based on morphological criteria. Colored rectangular borders denote the diagnostic class assigned during annotation (NILM, ASC-US, LSIL, HSIL). A custom QuPath script was used to export annotated regions at single-cell level and organize them into class-specific directories for model training.

*Annotation:* Due to the gigapixel resolution of WSIs, direct end-to-end processing of entire slides is computationally infeasible [22]. To enable efficient deep learning, we therefore adopted an annotation-driven, cell-centered patch learning strategy. Rather than uniformly tiling the whole WSI, training samples were generated exclusively around diagnostically relevant cellular targets to maximize the signal-to-noise ratio and to minimize the inclusion of background, mucus, and other non-informative regions. Specifically, each input sample consisted of a 224 × 224-pixel RGB patch extracted centered on a manually annotated target cell (or on the centroid of a manually delineated cellular region), thereby standardizing the input size for CNN backbones [23] while preserving local morphological context. Here, the 224 × 224-pixel crop is intended as a standardized, cell-centered field-of-view that captures the diagnostically dominant cytomorphological features while minimizing non-informative background. During annotation, experts verified that the extracted crop enclosed the target cell’s nuclear morphology and sufficient immediate context for Bethesda categorization. This framing supports consistent input dimensionality across CNN backbones while maintaining the key morphological evidence required for classification. Ground truth labels were established through a rigorous, expert-driven annotation workflow using QuPath (open-source; available at https://qupath.github.io/; accessed on 22 December 2025) [24] (Figure 2). Experienced cytopathologists manually reviewed WSIs at the target magnification and annotated individual diagnostic target cells according to established cytomorphological criteria, including nuclear size and contour irregularity, chromatin texture, nucleus-to-cytoplasm ratio, and cytoplasmic features. Each annotated target was definitively assigned to one of four diagnostic categories according to the Bethesda System for Reporting Cervical Cytology: NILM, ASC-US, LSIL, or HSIL [3]. To ensure label fidelity particularly for borderline morphologies (e.g., ASC-US versus LSIL) we implemented a multi-rater double-read and adjudication workflow. Initial annotations were produced by a primary rater and independently reviewed by a second board-certified pathologist. In case of disagreement, labels were resolved during a consensus session, where consensus could not be reached immediately, a final arbitration discussion was performed to assign a single definitive label. Where feasible, inter-rater agreement was quantified on a representative subset of WSIs using weighted Cohen’s κ [25], and all labels used for model training corresponded to the consensus outcome. Following annotation, a custom QuPath script was used to export annotated samples at the single-cell level. For each annotated cell/region, a 224 × 224-pixel patch centered on the annotation was extracted and stored in class-specific directories, ensuring a deterministic mapping between expert labels and model inputs. Importantly, this manual patch export was performed exclusively for ground-truth construction and controlled benchmarking, where expert-defined, cell-centered crops were required to ensure maximal label fidelity and to prevent automation-induced bias in sample selection. Consequently, manual patch extraction is not required for inference with the trained CNN models, as the classifier operates solely on standardized 224 × 224 RGB inputs and can therefore be applied to automatically generated cell-centered crops. In the prospective deployment workflow on WSIs, such crops are generated automatically by the preprocessing pipeline via systematic tiling (1024 × 1024) and candidate localization, thereby enabling fully automated slide processing while maintaining the decoupled two-stage design (localization and classification).

Importantly, this workflow relied entirely on human expertise and intentionally avoided upstream automated cell detection or semi-automated candidate generation, thereby minimizing automation-induced bias in ground truth construction and providing high-quality supervision for deep learning. Accordingly, segmentation errors cannot propagate into the reported classifier performance, since no automated segmentation was used to generate or select the evaluation samples. This design isolates classification benchmarking from localization quality and prevents confounding of test metrics by upstream segmentation variability.

*Patch Extraction:* WSIs represent digital scans of pathological tissue samples at gigapixel resolution. Due to the substantial data volume, these images cannot be processed directly by standard Convolutional Neural Networks (CNNs) without exceeding memory and processing constraints. Therefore, dividing high-resolution images into manageable subunits (patching) is a mandatory preprocessing step in deep learning-based cytological analysis [22]. In this study, a systematic patching and tissue detection procedure was applied to the digital cervical cytology slides. To facilitate analysis under computational resource constraints, each WSI was tessellated into fixed-size square patches of 1024 × 1024 pixels. The extraction process was executed using a raster-scan logic originating from the top-left corner of the image. To prevent data redundancy and enhance computational efficiency, a non-overlapping grid strategy was employed. This procedure was implemented in Python utilizing the OpenSlide (open-source; https://openslide.org/; accessed on 13 February 2026) [26], NumPy (open-source; https://numpy.org/; accessed on 13 February 2026) [27], and the Python Imaging Library via Pillow (open-source; https://python-pillow.org/; accessed on 13 February 2026), performed at the highest magnification level (Level 0) of the slides. As a result of this strategy, an average of 600 qualified patches was obtained per WSI. The tissue detection module provided a reduction in total data volume of approximately 40–60%.

*Stain Normalization:* Scanner variability results in significant differences in color tones, contrast, and staining intensity, which can degrade model performance (domain shift) [28]. Variations in staining protocols (e.g., Papanicolaou stain intensity) and scanner color calibration create a “color domain shift” that can mislead CNNs [29]. To mitigate this, we applied Reinhard Stain Normalization [30]. Reinhard normalization was applied as a baseline color harmonization step to reduce gross staining color variability. We emphasize that this preprocessing does not constitute a learned domain adaptation method. Rather, it provides a transparent and reproducible baseline upon which we evaluate the impact of multi-source training. Learned domain adaptation strategies are not implemented in the current study and are left for future work.

The RGB images are first converted into the LAB color space, where L represents luminance, and A and B represent chromaticity components and statistically align the mean and standard deviation of each channel to a target reference image. This decorrelates color information from intensity. The core operation involves transferring the statistical distribution (mean and standard deviation) of a reference target image to the source image. For each channel (L, A, B), the normalization is defined as:

(1) $$I_{norm}=\frac{I_{source}-\mu_{source}}{\sigma_{source}}\times \sigma_{target}+\mu_{target}$$

**Figure 3:**
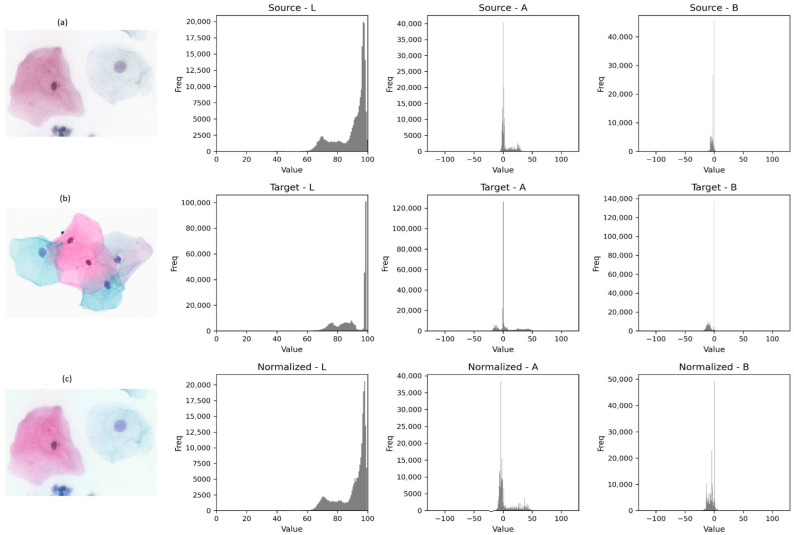
Visualization of the Reinhard stain normalization pipeline. The first column displays the (**a**) original Source image, (**b**) the reference Target image, and the (**c**) final Normalized output. The subsequent columns illustrate the corresponding pixel intensity histograms for the L (Lightness), A (Red–Green), and B (Blue–Yellow) channels in the LAB color space. As demonstrated, the normalization algorithm aligns the statistical distribution (mean and standard deviation) of the source image to match the target reference, effectively mitigating scanner-induced color variability. Differences in cell coloration across (**a**–**c**) represent staining/scanner-related appearance variability (domain shift) and its reduction after Reinhard normalization, colors are illustrative and not class labels.

Here, the vectors *μ* (mean) and *σ* (standard deviation) are the critical parameters. By aligning the *μ* and *σ* vectors of the input patches to those of a standardized reference slide, we force the neural network to focus on structural morphology (chromatin texture, nuclear-to-cytoplasmic ratio) rather than being biased by vendor-specific color casts (e.g., darker purple from Scanner A vs. lighter blue from Scanner B). This harmonization step minimizes vendor-specific color variations while preserving cytological structures (Figure 3).

**Figure 4:**
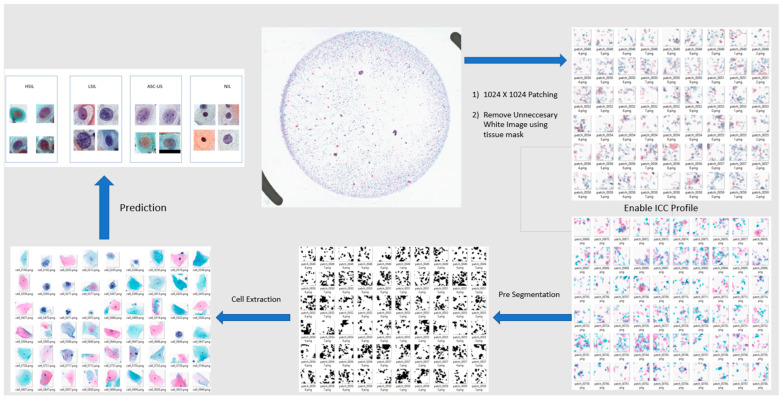
Overview of the preprocessing and inference pipeline for cervical cytology WSIs. A WSI is first partitioned into 1024 × 1024 patches and non-informative white background is removed using a tissue mask. Color normalization is then applied using an ICC-guided reference profile. Differences in cell coloration illustrate stain-scanner-induced appearance variability and its reduction after ICC-guided color correction, colors are illustrative and not class labels. The normalized patches undergo a pre-segmentation step to generate cell candidate regions, followed by cell extraction. The resulting cell-centered image tiles are subsequently fed into the trained deep learning classifier to produce class predictions.

An overview of the end-to-end preprocessing and inference workflow, from WSI patch extraction and ICC-guided color correction to pre-segmentation, cell extraction, and final classification is summarized in Figure 4.

### 2.3. Cellular Segmentation Strategy

**Figure 5:**
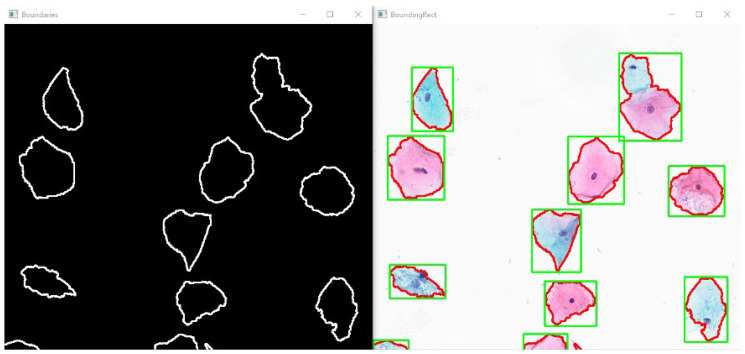
Visualization of cellular segmentation and object localization. This illustration depicts the outcome of the segmentation process aimed at isolating individual cytological structures from the background. The left panel, Boundaries, displays the extracted binary object contours against a black background. The right panel, BoundingRect, demonstrates the overlay of these segmentation contours (red lines) onto the original microscopic brightfield image. Additionally, the computed bounding boxes (green frames) are presented, which are utilized for subsequent object detection or cropping steps. Red outlines indicate the segmentation contours, and green rectangles denote the extracted bounding boxes, the underlying cell coloration is due to staining and does not represent class labels.

In the domain of automated cervical cytology analysis, segmentation constitutes a pivotal preprocessing stage aimed at accurately delineating cellular structures, specifically the nucleus and cytoplasm from the complex background inherent in Whole Slide Image (WSI) data [31]. These images are typically gigapixel-scale, often exceeding dimensions of 100,000 × 100,000 pixels, and comprise a heterogeneous mixture of thousands of epithelial cells, inflammatory infiltrates, overlapping clusters, variable staining intensities, and diverse artifacts. In the absence of a robust segmentation protocol, the high dimensionality and unstructured nature of raw WSI data can detrimentally impact classification model performance, impose prohibitive memory and efficiency constraints, and obscure the interpretability of model outputs [32]. In this work, segmentation is implemented as a candidate localization and cropping module for whole-slide inference rather than as an integrated learning component. The classifier is trained and evaluated on manually annotated, cell-centered image patches exported from QuPath, ensuring that feature learning is based on expert-defined ground truth rather than automatically generated masks. Therefore, segmentation does not contribute to the optimization objective and does not affect the formal test metrics of the classification model. This modular design avoids error propagation from imperfect segmentation to classification and improves robustness in heterogeneous, real-world WSI settings. Although WSI decomposition into 1024 × 1024 tiles may create boundary cases in which nuclei are partially visible within a single tile, segmentation in our pipeline is used as a localization preprocessing step, while diagnostic inference is defined at the WSI level. To minimize missed detections near tile borders, the slide is analyzed using boundary-shifted tile coverage (neighboring tile regions), such that cells at one tile edge are captured in adjacent analysis regions. Consequently, the segmentation approach employed in this study addresses three fundamental objectives. Firstly, the spatial isolation of diagnostically significant cells from vast, non-informative background regions to reduce computational noise. Secondly, the disentanglement of overlapping cellular structures, particularly within dense cell clusters, to ensure discrete analysis. Thirdly, the quantifiable representation of local morphological attributes such as shape, size, texture, and the nucleus-to-cytoplasm ratio for the downstream classification architecture. Ultimately, this structured segmentation process serves as a prerequisite for establishing an automated decision-support pipeline capable of reliably categorizing Pap smear images into the four standard diagnostic groups: NILM, ASC-US, LSIL, and HSIL. By converting raw pixel data into defined cellular objects, the system effectively bridges the gap between low-level image processing and high-level diagnostic reasoning. The cellular segmentation and object localization outputs used for subsequent cropping are illustrated in Figure 5.

### 2.4. Data Augmentation

**Figure 6:**
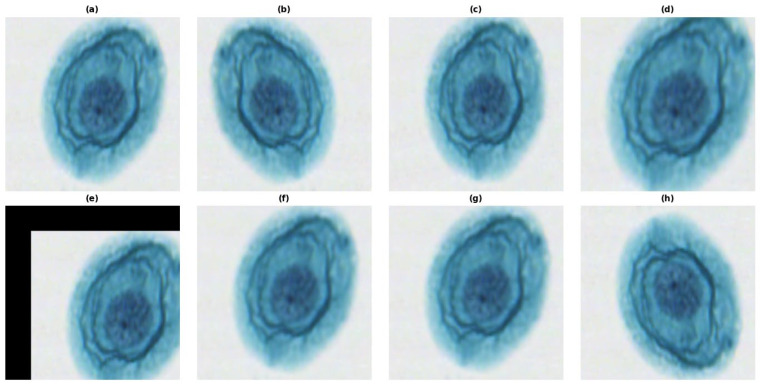
Overview of the normalization pipeline and preprocessing steps. During model training, real-time data augmentation was applied consistently across all evaluated CNN architectures (ResNet50, EfficientNet, and VGG16) to improve generalization. (**a**) Original unprocessed patch (224 × 224 px). (**b**) horizontal flip (**c**) Rotation up to ±15 degrees (**d**) Zooming up to %10 zoom out or %50 zoom in (**e**) Random cropping and translation up to ±10%, excluding darker border regions during training (**f**) Blurring, gaussian blurring with a kernel size randomly selected between 3 × 3 and 5 × 5 (**g**) Defocusing, motion blur with a randomly oriented kernel of up to 5 pixels. (**h**) vertical flip. These augmentation strategies were used to artificially increase the diversity of the training data and to improve the generalization performance of the ResNet50 model.

Deep learning models require large volumes of data to achieve translational invariance [33]. Furthermore, medical datasets inherently suffer from class imbalance [34], where negative samples (NILM) vastly outnumber pathological cases (HSIL/ASC-US). Ambiguous or borderline cases were reviewed by two board-certified pathologists to minimize inter-observer variability. We employed data augmentation for two purposes, first to simulate real-world acquisition artifacts (e.g., varying sample orientation, slight out-of-focus blurring). Secondly, to artificially oversample the minority classes, thereby preventing the model from converging to a trivial solution (predicting “Normal” for everything). The target image was selected by a pathologist representing the optimal staining quality. Geometric Transformations, random rotations (±15°) and horizontal flips were applied to enforce rotational invariance, as cell orientation is biologically irrelevant. Zooming scale 0.9–1.1 simulates slight variations in scanner magnification factors. Photometric distortions (Gaussian Blurring) were applied to mimic focus artifacts common in high-throughput scanning. All these synthetic augmentation techniques (rotation, Gaussian noise [35]) were applied to minority classes (ASC-US, LSIL) to address class imbalance. The final dataset was partitioned into training (80%), validation (10%), and testing (10%) sets. The primary rationale for allocating 80% of the dataset to the training phase is to mitigate statistical bias and enhance the model’s predictive accuracy through exposure to a broader distribution of data. A fundamental inverse correlation exists between the volume of the training samples and the propensity for overfitting; as the dataset size increases, the model’s ability to capture underlying patterns rather than noise is mathematically fortified [36]. Furthermore, the validation set is utilized as an independent proxy for hyperparameter optimization and model selection, while the final test set serves as a rigorous benchmark to evaluate the model’s generalization capability on entirely unseen instances, thereby ensuring the empirical validity of the results. To ensure clinical fidelity, the normalized images underwent a qualitative assessment by board-certified pathologists. This visual inspection confirmed that the Reinhard algorithm successfully harmonized background and cytoplasmic intensities across the multi-center dataset without altering critical diagnostic features. Specifically, the fine-grained nuclear chromatin patterns and hyperchromasia, which are pivotal for distinguishing malignancy, remained structurally intact, ensuring that the preprocessing step did not induce any loss of biological information (see Figure 6 for an overview of the data normalization).

### 2.5. Deep Learning Architecture and Training Strategy

To identify the optimal computational architecture for handling the high variability of the multi-center dataset, we conducted a comparative study using three state-of-the-art Convolutional Neural Networks (CNNs). All models were utilized within a transfer learning framework [37], initialized with weights pre-trained on the ImageNet dataset. This approach leverages learned feature representations (e.g., edges, textures) from large-scale data, accelerating convergence and improving robustness on medical images. We systematically evaluated the following backbone architectures to assess the trade-off between classification accuracy, model complexity, and computational efficiency: ResNet50 [38] was selected for its utilization of residual skip connections, which address the vanishing gradient problem and facilitate the training of deeper networks comprising 50 layers, effectively capturing hierarchical features in complex cytological structures. EfficientNetB0 [39] was included for its innovative compound scaling method that uniformly scales network width, depth, and resolution to maximize accuracy while minimizing parameter counts. In contrast, the classic VGG16 [40] architecture served as a robust baseline for feature extraction; although computationally expensive, it is characterized by a uniform stack of convolutional layers utilizing small 3 × 3 receptive fields. To ensure a rigorous and fair comparison across these diverse backbones, the original fully connected classification layers of all three architectures were removed and replaced with an identical custom classification head. This standardized architecture comprises a Global Average Pooling layer [41] to reduce spatial dimensions and minimize overfitting, followed by a fully connected Dense Layer with 256 units and ReLU activation. A Dropout Layer (rate = 0.5) was integrated for regularization, leading to a final Softmax Layer that outputs probabilities for the four diagnostic classes (NILM, LSIL, HSIL, ASC-US). Based on the preliminary comparative analysis (detailed in Section 3), ResNet50 demonstrated the superior balance of sensitivity and specificity for this specific multi-center domain. Consequently, it was selected as the primary backbone for the subsequent ablation studies regarding scanner variability.

**Figure 7:**
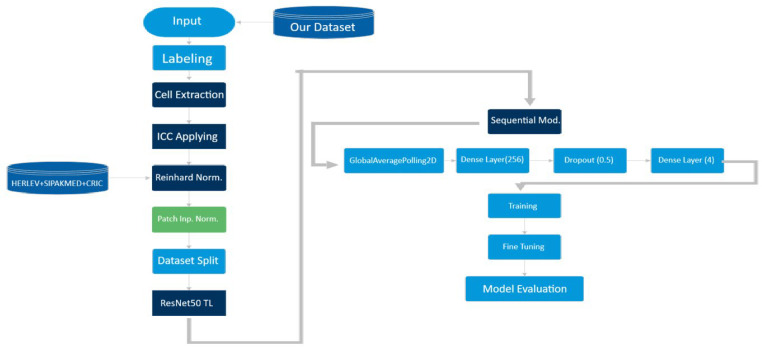
Schematic block diagram of the proposed deep learning framework. The pipeline begins with the input of preprocessed 224 × 224-pixel patches. The ResNet50 backbone (pre-trained on ImageNet) serves as the primary feature extractor. Here, ‘Sequential Mod.’ denotes the Keras (TensorFlow) Sequential API used to implement the classifier head as a linear stack of layers appended to the pretrained backbone (ResNet50 → Global Average Pooling → fully connected layers → softmax). The extracted feature maps are then processed by the custom classification head, which consists of a Global Average Pooling layer to reduce dimensionality, followed by a fully connected Dense layer (256 units, ReLU activation), a Dropout layer (rate = 0.5) for regularization, and a final Softmax layer. This architecture outputs the probability distribution for the four diagnostic classes: NILM, ASC-US, LSIL, and HSIL.

We employed a transfer learning approach using ResNet50 as the backbone architecture due to its superior parameter efficiency and residual learning capabilities, which effectively mitigate the vanishing gradient problem (Figure 7). The classification head was implemented using the Keras (TensorFlow) Sequential API as a linear stack of layers appended to the pretrained backbone. Specifically, a pretrained ResNet50 backbone (with frozen weights during the initial training stage) was followed by Global Average Pooling and fully connected layers culminating in a softmax output for four-class prediction.

**Figure 8:**
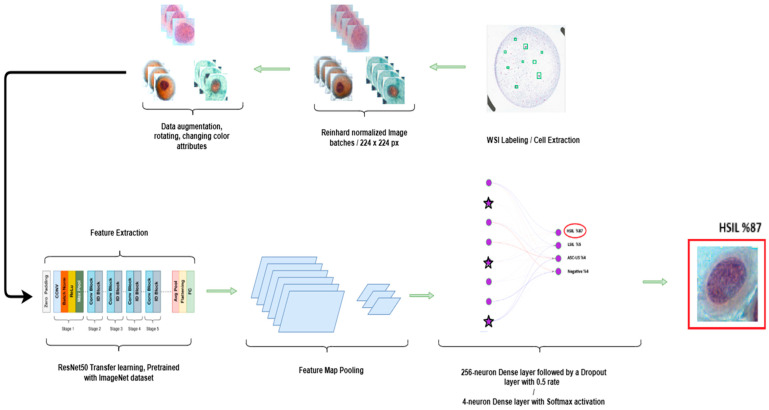
Detailed architecture of the proposed cervical cell classification model. The system utilizes a ResNet50 backbone (pre-trained on ImageNet) as the primary feature extractor. The custom classification head consists of: (1) a Global Average Pooling layer to reduce spatial dimensions; (2) a Dense layer with 256 units and ReLU activation for feature transformation; (3) a Dropout layer (rate = 0.5) to mitigate overfitting; and (4) a final Softmax output layer that predicts the probability for the four diagnostic categories: HSIL, LSIL, ASC-US, and Negative (NILM). Colors are used solely to visually distinguish pipeline stages (augmentation, normalization, feature extraction, and classification) and do not encode additional quantitative information.

The model architecture was modified for the specific task of 4-class cervical cell classification (Figure 8). The convolutional base of ResNet50, pre-trained on ImageNet, was used to extract high-level features (textures, edges).

### 2.6. Experimental Setup and Training Protocol

The proposed deep learning framework training and evaluation were performed on an Apple MacBook Pro (Apple Inc., Cupertino, CA, USA) featuring an M3 Max SoC (14-core CPU/32-core integrated GPU) with 1 TB SSD storage. Models were implemented using TensorFlow (Google LLC, Mountain View, CA, USA) [42], version 2.16.2 under macOS (Sonoma 14.2.1) where supported hardware acceleration was enabled via Apple’s GPU compute backend. To optimize the model, we used the Adam optimizer [43] due to its adaptive learning rate capabilities, employing a batch size of 8 to balance memory constraints with gradient stability, while categorical cross-entropy was selected as the loss function for the multi-class classification task. To mitigate overfitting during training, early stopping [44] was applied with a patience of 10 epochs. Regarding data partitioning, the consolidated dataset was divided into Training (80%), Validation (10%), and Testing (10%) subsets to enable a rigorous assessment of generalization. This split ratio was motivated by the inherent class imbalance in clinical Pap smear data, where negative samples (NILM) substantially outnumber pathological categories such as HSIL or ASC-US. Consequently, stratified sampling was applied to preserve class proportions across subsets and reduce representation bias. To prevent patch-level information leakage, dataset splitting was performed using grouped stratification with disjoint grouping keys [45]. Patient IDs were used as the grouping key for the proprietary cohort (416 WSIs), whereas slide/image identifiers were used as the grouping key for public datasets without patient IDs. Split integrity was subsequently audited using WSI/slide identifiers by computing the set intersection between splits (Train∩Val, Train∩Test, Val∩Test), which was zero in all cases. To provide dataset-level transparency of the data split, Table 1 summarizes the number of extracted cell-centered patches assigned to training, validation, and test subsets for each dataset source. To prevent information leakage, data partitioning was performed at the WSI level whenever slide identifiers were available. All cell-centered patches extracted from the same WSI were assigned to the same subset (training, validation, or test). For the proprietary cohort, WSIs were split prior to patch extraction, ensuring that no slide contributes patches to multiple subsets. All reported quantitative results in this study are computed on 224 × 224 cell-centered patches. We do not perform slide-level label aggregation (e.g., MIL) and therefore do not report WSI-/patient-level screening endpoints.

Within this protocol, the Training Set was utilized to update model weights and expose the network to sufficient morphological variability of minority classes, whereas the Validation Set served for hyperparameter tuning and monitoring early stopping criteria. The Test Set was maintained strictly as a held-out subset for the final performance evaluation on unseen clinical data. To maximize classification performance, a two-stage transfer learning approach was adopted [46]. In Phase 1 (Feature Preservation), the ResNet50 backbone was frozen, and only the custom classification head was trained for 20 epochs with an initial learning rate of 1 × 10^−4^. This allowed the classifier to adapt to cytology features without degrading the pre-trained ImageNet weights. Subsequently, in Phase 2 (Fine-Tuning), the top 2 layers of the backbone were unfrozen and fine-tuned with a reduced learning rate of 1 × 10^−5^.

### 2.7. Evaluation Metrics

To rigorously assess model performance in a multi-class setting characterized by class imbalance, we employed the following metrics. All values were calculated using a macro-averaging strategy to ensure that minority classes (such as HSIL and ASC-US) contribute equally to the final score, preventing results from being skewed by the majority NILM class. The ratio of correctly predicted observations to the total number of observations is accuracy. F1 Score is the harmonic mean of precision and recall, calculated for each class and then averaged. This serves as our primary metric for assessing robustness. Calculated for each class against all other classes to measure the model’s ability to correctly reject non-target categories (e.g., the specific ability to distinguish non-HSIL samples).

$$F1=\frac{1}{N}\sum_{i=1}^{N}\frac{2.{Precision}_{i}\;.\;{Recall}_{i}}{{Precision}_{i}+{Recall}_{i}}$$

## 3. Results

In this section, we present the quantitative evaluation of the proposed multi-center deep learning framework. The analysis focuses on three key aspects: (1) the training stability and convergence behavior, (2) the comparative performance against other state-of-the-art architectures, and (3) the impact of including proprietary multi-scanner data on domain generalization.

### 3.1. Training Stability and Convergence Analysis

**Figure 9:**
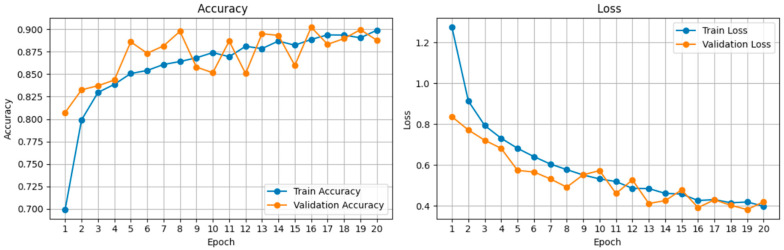
Evaluation of Model Training and Validation Performance via Learning Curves. The training dynamics of the proposed deep learning classifier were monitored over 20 epochs to assess convergence stability and generalization capability. Accuracy Analysis (**Left**): The training accuracy shows a rapid initial ascent, asymptotically stabilizing at approximately 90%. The validation accuracy closely tracks the training curve throughout the process, indicating that the model successfully captured the underlying biological features without significant overfitting. Loss Function Analysis (**Right**): Both training and validation loss exhibit a consistent, monotonic decrease, confirming the efficacy of the optimization strategy. The minimal divergence between the two loss curves suggests that the implemented data augmentation and stain normalization techniques effectively regularized the model, ensuring robust performance across varying data distributions.

The learning dynamics of the ResNet50 model over 20 (this value can be reduced by Early Stopping) epochs are illustrated in Figure 9. The training and validation loss curves demonstrate a consistent downward trajectory, stabilizing around epoch 15. Notably, the gap between training and validation loss remains minimal (<0.5), indicating that the employed regularization techniques (Dropout, Data Augmentation) effectively prevented overfitting despite the high complexity of the feature space. The accuracy curves show a corresponding logarithmic increase, reaching a plateau at 89% validation accuracy. This convergence behavior confirms that the learning rate schedule (Phase 1: 1 × 10^−4^, Phase 2: 1 × 10^−5^) successfully balanced rapid feature adaptation with stable fine-tuning.

### 3.2. Comparative Analysis of Deep Learning Architectures

To evaluate the efficacy of different convolutional neural network backbones, a comprehensive performance benchmarking was conducted on the multi-center test set. Table 2 summarizes the quantitative results for the ResNet50, VGG16, and EfficientNetB0 architectures across three key metrics Accuracy, Sensitivity, and F1-score.

Among the evaluated models, ResNet50 demonstrated superior performance, achieving the highest scores across all metrics with a consistent value of 0.91 for accuracy, sensitivity, and F1-score. This indicates that the residual learning framework of ResNet50 effectively captured the relevant features within the multi-center dataset, offering the most robust generalization capabilities. The VGG16 architecture yielded competitive results, closely trailing ResNet50. While it matched the sensitivity of the optimal model (0.91), it exhibited a marginal decrease in overall accuracy and F1-score, both recording 0.90. This suggests that while VGG16 remains a strong candidate for sensitivity-critical tasks, ResNet50 offers a slight advantage in overall predictive precision. Conversely, EfficientNetB0 showed the lowest performance among the tested backbones, with all three metrics stabilizing at 0.86. The comparative gap between EfficientNetB0 and the heavier architectures (ResNet50 and VGG16) suggests that, for this specific multi-center application, the model complexity and depth provided by ResNet50 are necessary to maximize classification performance. Based on this benchmarking analysis, ResNet50 was identified as the most suitable backbone for the proposed framework.

### 3.3. Classification Performance and Confusion Matrix

The diagnostic performance of the trained ResNet50 Convolutional Neural Network was rigorously evaluated using independent validation and test datasets, each comprising 10% of the total data volume. The following sections detail the model’s ability to classify cytopathological instances within a multi-center, mixed dataset environment. The analysis of the validation dataset (Table 3) yielded an overall classification accuracy of 92%. The model demonstrated exceptional precision in identifying the NILM (Negative for Intraepithelial Lesion or Malignancy) class, achieving a precision of 0.97 and an F1-score of 0.95. This high specificity is critical for minimizing false positives in automated screening workflows. For the pathological categories, the model maintained robust discriminative power: HSIL achieved a high recall (sensitivity) of 0.90, emphasizing the model’s clinical reliability in detecting high-risk cases. ASC-US and LSIL and recorded F1-scores of 0.85 and 0.86, respectively, demonstrating stable performance even in cases with subtle morphological variations.

To assess the model’s generalization performance on entirely unseen data, a final evaluation was conducted on the test dataset (Table 4). The model exhibited remarkable stability, maintaining an overall accuracy of 91%. The key findings are as follows: NILM class confirmed superior performance with a persistent F1-score of 0.95. ASC-US class demonstrated a notable sensitivity (recall) of 0.92, indicating successful feature extraction despite the inherent ambiguity often associated with borderline morphologies. Precision for LSIL remained high at 0.90, while the F1-scores for both classes stabilized at 0.84, reflecting the model’s consistent predictive fidelity.

The macro and weighted averages for precision, recall, and F1-score across both datasets ranged between 0.87 and 0.92. The minimal variance (Δ < 1%) between validation and test metrics suggests that the ResNet50 architecture, supported by our data augmentation and stain normalization pipeline, successfully mitigated the risk of overfitting.

**Figure 10:**
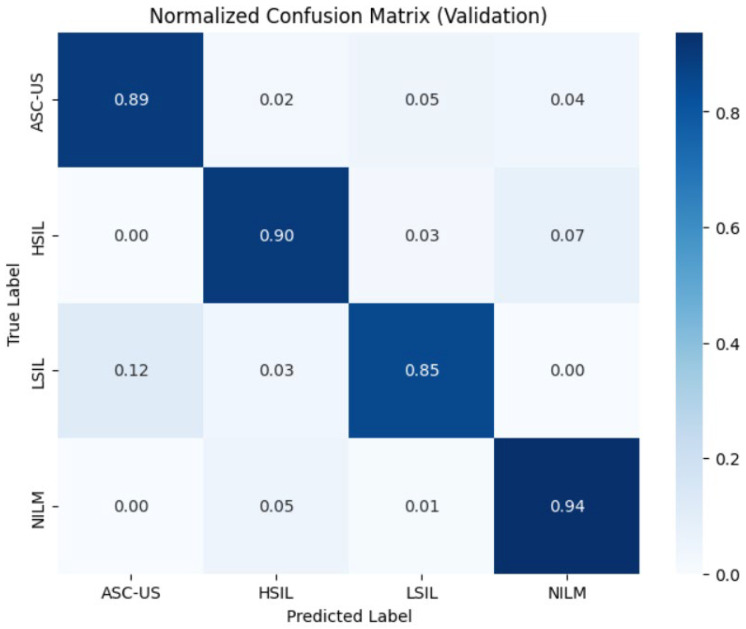
Normalized confusion matrix illustrating classification performance on the independent validation dataset. The matrix depicts the deep learning model’s ability to differentiate between the four Bethesda diagnostic categories (NILM, LSIL, HSIL, ASC-US). The diagonal elements represent the correct classification rates (class-wise sensitivity), while off-diagonal elements quantify misclassifications between categories.

**Figure 11:**
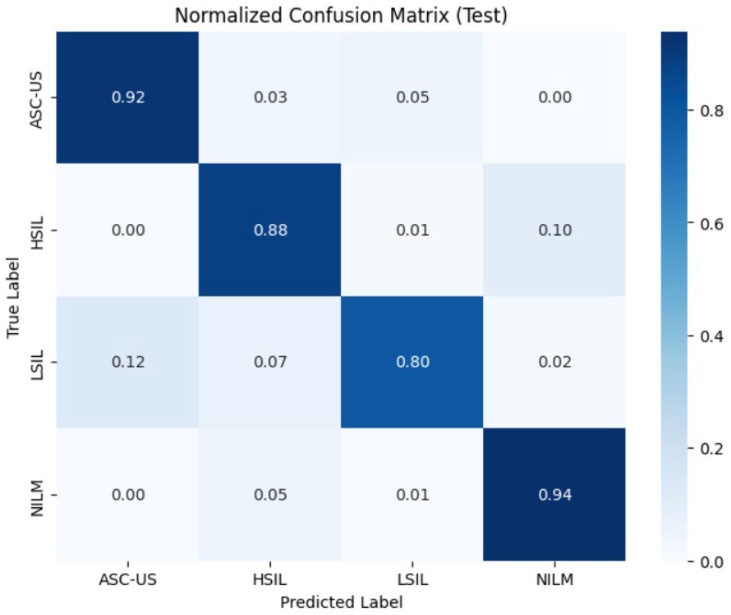
Normalized confusion matrix illustrating classification performance on the independent test dataset. The matrix depicts the deep learning model’s ability to differentiate between the four Bethesda diagnostic categories (NILM, LSIL, HSIL, ASC-US). The diagonal elements represent the correct classification rates (class-wise sensitivity), while off-diagonal elements quantify misclassifications between categories.

To evaluate the diagnostic precision of the ResNet50 model at a granular level, the normalized confusion matrices for both the validation and test datasets were analyzed (Figure 10 and Figure 11). The results demonstrate high system reliability in identifying physiological cellular patterns and a clinically relevant ability to differentiate between various lesion severities. The model exhibited the highest robustness in identifying normal findings (NILM), with a consistent sensitivity of 94% across both datasets. Only 5% of NILM cases were incorrectly classified as HSIL. For the most clinically critical category, HSIL (High-Grade Squamous Intraepithelial Lesion), the model achieved a correct classification rate of 90% in the validation dataset and 88% in the test dataset. The misclassification rate of HSIL as NILM (false-negative) ranged between 7% and 10%, representing a clinically significant parameter that underscores the need for further model optimization in high-risk early detection. The ASC-US category showed a sensitivity of 89% in the validation set, which increased to 92% in the test set. Nevertheless, a systematic overlap with LSIL findings was observed. Sensitivity for LSIL lesions was 85% in validation and decreased to 80% in the test set. Notably, there was a significant confusion rate with ASC-US, reaching 12% in the test dataset. Beyond overall accuracy and macro-F1, we performed a screening-oriented error analysis emphasizing the asymmetric clinical risk of false negatives, and we therefore report HSIL(+) sensitivity/recall, FNR, NPV, and the critical HSI → NILM undercall rate in addition to conventional metrics. Table 5 reports class-wise sensitivity (recall), under-call rates (1 − recall), and clinically critical misclassification rates (to NILM) on the independent test set. Figure 11 indicates an HSIL sensitivity of 0.88 on the independent test set, with the predominant HSIL error mode being misclassification as NILM (0.10), whereas grade-adjacent confusions were rare (HSIL as LSIL: 0.01 or HSIL as ASC-US: 0.00). This pattern suggests that the model generally maintains a high threshold for high-grade abnormality, and that remaining false negatives are concentrated in the clinically most consequential direction. We therefore report HSIL recall and the HSIL to NILM rate explicitly as safety-relevant indicators and prioritize this error boundary for future optimization and triage calibration.

These results verify that the proposed framework achieves a high degree of empirical validity and provides a robust foundation for an automated clinical decision support system in digital cytopathology.

### 3.4. Impact of Multi-Source Training on Domain Generalization

The central hypothesis of this study is that training on diverse, multi-scanner data is essential for clinical robustness. To quantify this, we compared the performance of a ResNet50 model trained exclusively on harmonized public data against the proposed model trained on a comprehensive Multi-Source Dataset. Both models were evaluated using a proprietary test set containing real-world scanner artifacts to simulate clinical deployment. The generalizability of the proposed ResNet50 framework was evaluated through a rigorous Domain Shift Analysis (Table 6). This assessment quantifies the impact of data source diversity on model performance when encountering unseen scanner characteristics and staining protocols. The experimental results demonstrate that models trained on homogeneous, single-center datasets exhibit high internal accuracy but fail significantly when deployed across different domains.

HERLEV Baseline: While achieving a peak accuracy of 95.11% on its internal test set, the performance plummeted to 72.76% (Accuracy) and 0.71 (F1-score) when evaluated against the SIPaKMeD dataset.

SIPaKMeD Baseline: Similarly, a model trained exclusively on SIPaKMeD achieved near-perfect internal metrics (98.77% Accuracy; 0.99 F1-score) but showed a sharp decline in generalization to the HERLEV domain, with the F1-score dropping to 0.76.

These findings highlight a classic overfitting to domain-specific features, such as vendor-specific color profiles and lighting conditions, rather than invariant morphological characteristics. The integration of diverse datasets (HERLEV, SIPaKMeD, CRIC, and proprietary Multi-Center data) yielded a model with superior cross-domain resilience. The comprehensive model by proposed Multi-Center Model achieved a balanced performance across all centers, maintaining an Accuracy of 91.00% and an F1-score of 0.91 on the heterogeneous multi-center test set.

These findings suggest that incorporating multi-scanner variability (e.g., Leica, Hamamatsu, 3DHistech) during training can be important for improving robustness and for moving toward clinically applicable cervical cytology AI systems. The proposed multi-source approach successfully bridged the domain gap, restoring the F1-score from 0.78 (Baseline) to 0.92.

## 4. Discussion

### 4.1. Addressing the Domain Shift in Digital Cytopathology

The primary challenge impeding the widespread clinical adoption of AI in cervical cancer screening is the domain shift phenomenon, where models trained on curated datasets fail to generalize to real-world clinical data. Our study directly addressed this by moving beyond standard public repositories. While previous studies have achieved high accuracy on homogeneous datasets like Herlev or SIPaKMeD [7,8], they often struggle when applied to images acquired from different scanners or staining protocols. By integrating a proprietary dataset from two distinct medical centers and three different scanner vendors, our ResNet50-based framework demonstrated significant robustness. This suggests that data diversity during training is as critical as the choice of model architecture.

### 4.2. Architecture Performance and Interpretability

Among the evaluated architectures, ResNet50 consistently outperformed VGG16 and EfficientNetB0. This aligns with findings in histopathology, where residual connections facilitate the learning of deep, hierarchical features such as chromatin texture and nuclear membrane irregularities without suffering from the vanishing gradient problem. The superior specificity of ResNet50 (0.91) is particularly clinically relevant, as it reduces false positives, thereby minimizing unnecessary anxiety for patients and reducing the workload for pathologists reviewing normal cases.

To contextualize the performance of our proposed multi-center ResNet50 framework, we compared our results with recent state-of-the-art (SOTA) studies in cervical cytology automation. As summarized in Table 3, numerous studies have reported exceptional accuracies ranging from 95% to 98% [35,36]. However, a critical analysis reveals that these benchmarks are predominantly established on the Herlev or SIPaKMeD datasets under idealized conditions. For instance, Rahaman et al. [5] achieved an accuracy of 98.3% using a DeepCervix framework. While impressive, this performance was evaluated on manually cropped, centered single cells with clean backgrounds, which does not reflect the complexity of whole-slide screening. Similarly, Plissiti et al. [7] reported high efficacy using VGG-based features but restricted their evaluation to artifact-free fields of view. In contrast, our study accepts a slight trade-off in absolute numerical metrics to gain significant clinical translational value. While our achieved accuracy of 91.0% is marginally lower than the 98% reported on single-cell datasets, it was obtained on a multi-center WSI dataset containing scanner artifacts, overlapping cells, and inflammatory backgrounds.

This distinction is of paramount importance because models trained exclusively on pristine or idealized data sets frequently exhibit catastrophic failure when deployed within real-world pathology workflows due to inherent domain shifts. Consequently, our results demonstrate a superior level of robustness, providing empirical evidence that the proposed framework maintains high diagnostic reliability even when confronted with realistic scanner variations and significant pre-analytical heterogeneity [47,48].

While Table 7 provides comparison with prior cell-/patch-level benchmarks, we acknowledge that recent cervical cytology research has increasingly shifted toward WSI-/slide-level evaluation and large multi-center validation, where clinically actionable outputs require aggregation of instance-level evidence. Accordingly, Table 8 summarizes representative WSI-level screening systems that explicitly address generalization under acquisition and center variability. Within this context, our study contributes to rigorous cell-level benchmarking and systematic robustness ablations across multi-source data combinations, and we position the proposed classifier as a modular component that can be integrated into end-to-end WSI screening pipelines with slide-level aggregation in future work.

### 4.3. Clinical Implications

The developed system is designed not to replace cytopathologists but to serve as a high-sensitivity pre-screening triage tool. By reliably identifying high-risk slides (HSIL/LSIL) and filtering out clear negatives, the algorithm allows pathologists to focus their attention on complex, ambiguous cases. The successful harmonization of data from private and public hospitals indicates that such a system could be deployed across institutions with different hardware setups, a prerequisite for scalable telediagnosis.

### 4.4. Limitations and Future Directions

Despite promising results, this study has limitations. First, while we addressed class imbalance via augmentation and class weighting, the number of HSIL cases remains lower than NILM, reflecting the natural prevalence of the disease. Second, although we included data from three scanners, validation on a wider range of devices (e.g., from additional vendors) would further confirm generalizability. Finally, our current model operates at the patch level; integrating a slide-level aggregation module to generate a whole-slide diagnosis score is a planned future improvement. While the proposed multi-center framework demonstrates significant robustness against scanner-induced domain shifts, this study is subject to certain limitations that delineate the scope for future research.

First, our framework was developed and validated exclusively on Liquid-Based Cytology samples (ThinPrep and BD SurePath). Unlike conventional Pap smears, LBC slides offer a monolayer distribution of cells with reduced background obscuration from blood or inflammation. In contrast, conventional smears are characterized by high degrees of cytological superposition (overlapping cells), uneven smear thickness, and scanning artifacts particularly at the smear edges where focus points are difficult to maintain. Consequently, the direct applicability of our model to conventional smears remains unproven. While parts of conventional slides could theoretically be analyzed for research purposes or data augmentation, the dense clustering and physical artifacts render them suboptimal for the current automated pipeline without additional pre-processing steps for cell dispersion.

Second, the current performance metrics are reported at the patch level (ROI). While high accuracy in classifying individual cellular regions is the fundamental prerequisite for automated screening, it does not constitute a final patient-level diagnosis. A clinical diagnosis requires the aggregation of thousands of patch predictions to generate a Whole Slide Image (WSI) inference. For instance, a single HSIL patch among thousands of normal patches should trigger an alert, whereas isolated ASC-US predictions might require a probabilistic threshold. In this study, we prioritized the development of a feature extractor that is invariant to scanner variability. Although multi-source training and baseline color normalization improved robustness in our patch-level experiments, state-of-the-art WSI screening systems increasingly rely on learned domain adaptation and test-time adaptation to handle large-scale multi-center variability. Integrating such approaches together with slide-level aggregation (MIL) represents an important next step toward end-to-end screening.

Future iterations of this framework will address these challenges by extending the dataset to include annotated conventional Pap smears to test cross-modal generalization and implementing a slide-level decision matrix that mimics the pathologist’s workflow of screening and final reporting.

## 5. Conclusions

This study demonstrates a robust deep learning framework for the automated classification of liquid-based Pap smear cytology. By constructing a comprehensive dataset that integrates major public repositories with proprietary multi-center data digitized across diverse scanning systems, we successfully mitigated the critical challenge of scanner-induced domain shifts. Our ResNet50-based architecture achieved high-performance metrics, reaching an accuracy of 91%, which underscores that exposure to hardware-related variability during the training phase is a mandatory prerequisite for clinical reliability. These findings provide a scalable foundation for developing vendor-agnostic AI diagnostic tools. Such systems hold the potential to significantly support pathologists, reduce diagnostic backlogs, and standardize cervical cancer screening accuracy on a global scale.

## Figures and Tables

**Table 1:** Dataset-level distribution of patches across training, validation, and test subsets: All samples are 224 × 224 cell-centered patches. Dataset sources differ in class coverage (CRIC provides HSIL and NILM, SIPaKMeD/Herlev contribute LSIL and ASC-US).

Dataset	Training (*n*)	Validation (*n*)	Test (*n*)	Total (*n*)
Herlev	183	22	26	231
SIPaKMeD	1300	155	163	1618
CRIC	7030	873	879	8782
Proprietary (our dataset)	344	40	55	439

**Table 2:** Performance benchmarking of different backbones on the multi-center test set.

Method	Accuracy	Sensitivity	F1-Score
ResNet50	0.91	0.91	0.91
VGG16	0.90	0.91	0.90
EfficientNetB0	0.86	0.86	0.86

**Table 3:** Performance Metrics of the ResNet50-based Proposed Model on the Validation Dataset.

Method	Precision	Recall	F1-Score
ASC-US	0.83	0.89	0.86
HSIL	0.83	0.90	0.86
LSIL	0.86	0.85	0.85
NILM	0.97	0.94	0.95

**Table 4:** Performance Metrics of the ResNet50-based Proposed Model on the Test Dataset.

Method	Precision	Recall	F1-Score
ASC-US	0.81	0.92	0.86
HSIL	0.80	0.88	0.84
LSIL	0.90	0.80	0.84
NILM	0.97	0.94	0.95

**Table 5:** Clinically oriented error characterization on the independent test set (Figure 11). Critical FN to NILM” reports the fraction of cases in each true class that is predicted as NILM (screen-negative).

Classes	*n* (Test)	Sensitivity (Recall)	Under-Call Rate (1−Recall), x/*n* (%)	Critical Misclassifi- Cation * (to NILM) (%)	Clinical Impact
HSIL	215	0.88	26/215 (12.1%)	10%	CRITICAL RISK: High-grade lesion missed as healthy. Requires immediate biopsy.
LSIL	126	0.80	25/126 (19.8%)	2%	MODERATE RISK: Potential for progression; %2 missed, %12 downgraded to ASC-US.
ASC-US	91	0.92	7/91 (7.7%)	0%	LOW RISK: No lesions missed as NILM. 8% confused with LSIL/HSIL (Over-diagnosis).
NILM	691	0.94	41/691 (5.9%)	-	SPECIFICITY: 6% False Positive rate (unnecessary follow-up cost).

* Miss Rate (FN) = 1 − Recall.

**Table 6:** Impact of training data diversity on model generalization (Domain Shift Analysis).

Train Dataset	Test Dataset	Accuracy (%)	Sensitivity	Precision	F1-Score
HERLEV	HERLEV	95.11	0.91	0.96	0.93
	SIPaKMeD	72.76	0.73	0.79	0.71
SIPaKMeD	HERLEV	84.51	0.72	0.87	0.76
	SIPaKMeD	98.77	0.99	0.99	0.99
Herlev, SIPaKMeD, CRIC Herlev, SIPaKMeD, CRIC, Multi-Center DataSet	Herlev, SIPaKMeD, CRIC	94.26	0.92	0.92	0.92
	Herlev, SIPaKMeD, CRIC, Mutli-Center DataSet	91.00	0.91	0.91	0.91

**Table 7:** Comparison of the proposed framework with existing state-of-the-art methods.

Study (Reference)	Dataset Used	Method/Backbone	Accuracy	Limitation vs. Our Work
Rahaman et al. [5]	Herlev (Single Cell)	DeepCervix (Hybrid CNN)	98.3%	Tested on single cells only; no WSI artifacts.
Plissiti et al. [6]	SIPaKMeD (Clusters)	VGG19 + SVM	96.0%	Limited to one camera source; no scanner diversity.
Kurnianingsih et al. [42]	Herlev + Local	ResNet50	95.5%	Small dataset size; limited generalization.
Proposed Method	Multi-Center (Public + Proprietary)	ResNet50 (Transfer Learning)	91.0%	Validated on heterogeneous WSI data from 3 scanners.

**Table 8:** WSI-/Slide-level screening systems (state-of-the-art clinical evaluation paradigm).

Study	Data Level	Settings	Core Idea	Evaluation Highlights
Cheng et al., Nat Commun (2021) [10]	WSI-level	Large-scale WSI screening cohort	Deep learning WSI analysis with robustness focus across acquisition variability	Robust WSI screening performance reported—clinical screening framing
Zhu et al., Nat Commun (2021) [9]	Smear-level/large cohort cytology	>81k retrospective samples (TBS-based)	AI-assisted cytology aligned to Bethesda criteria	Large multicenter validation
STRIDE, Li et al., (2024) [14]	WSI-level	End-to-end WSI screening	Scalable learning combining patient-level labels with limited cell-level labels; robustness and interpretability	Designed to address annotation bottlenecks + domain variability
Smart-CCS/CCS-127K, Jiang et al. (2025) [15]	WSI-level	127,471 WSIs from 48 centers	Large-scale pretraining + test-time adaptation for generalizability	Multi-cohort eval; AUC ~0.95–0.97; sensitivity ~0.91 reported
Jiang et al., AI Review (2023) [4]	Review	2015–2023	Systematic review: cell identification → WSI analysis	Summarizes transition to WSI-level methods

## Data Availability

Publicly available datasets were analyzed in this study. This data can be found here [5,6,9]. Herlev Dataset http://mde-lab.aegean.gr/index.php/downloads/ (accessed on 4 March 2021). The single-cell Pap smear images are publicly available and were accessed through the Herlev University Hospital repository. SIPaKMeD Dataset https://www.cs.uoi.gr/~marina/sipakmed.html (accessed on 13 April 2021). The dataset comprising benign and dysplastic cell clusters was obtained from the SIPaKMeD Database available at the University of Ioannina repository. CRIC Cervix Dataset https://www2.decom.ufop.br/cric/cric-database/ (accessed on 23 October 2024). The liquid-based cytology images and whole-slide patches are available through the CRIC. The proprietary WSI dataset used in this study is not publicly available due to patient privacy and ethical restrictions. The patched single cell images, source code and trained models presented in this study are openly available on GitHub at https://github.com/users/gsava/projects/1, accessed on 31 December 2025.

## Abbreviations

The following abbreviations are used in this manuscript:

AI	Artificial Intelligence
ASC-US	Atypical Squamous Cells of Undetermined Significance
CCD	Charge Coupled Device
CNN	Convolutional Neural Network
CPU	Central Processing Unit
GPU	Graphics Processing Unit
HSIL	High-Grade Squamous Intraepithelial Lesion
ICC	International Color Consortium
LAB	CIELAB Color Space (Lightness, A, B channels)
LBC	Liquid-Based Cytology
LSIL	Low-Grade Squamous Intraepithelial Lesion
NA	Numerical Aperture
NILM	Negative for Intraepithelial Lesion or Malignancy
PAP	Papanicolaou
PIL	Python Imaging Library
ReLU	Rectified Linear Unit
ResNet	Residual Neural Network
ROI	Region of Interest
RGB	Reed, Green, Blue (Color Space)
SES	Socio- Economic Status
SoC	System on Cip
SSD	Solid State Drive
WSI	Whole Slide Imaging
WHO	World Health Organization
